# Insights from Eye Blinks into the Cognitive Processes Involved in Visual Word Recognition

**DOI:** 10.5334/joc.343

**Published:** 2024-01-17

**Authors:** Ronen Hershman, David L. Share, Elisabeth M. Weiss, Avishai Henik, Adi Shechter

**Affiliations:** 1 Department of Psychology, University of Innsbruck, Innsbruck, Austria; 2 Department of Learning Disabilities, Faculty of Education, University of Haifa, Haifa, Israel; 3 Edmond J. Safra Brain Research Center for the Study of Learning Disabilities, University of Haifa, Haifa, Israel; 4 Department of Psychology and The Zelman Center for Brain Science, Ben-Gurion University of the Negev, Beer-Sheva, Israel

**Keywords:** word recognition, reading, eye blinks, mental effort

## Abstract

Behavioral differences in speed and accuracy between reading familiar and unfamiliar words are well-established in the empirical literature. However, these standard measures of skill proficiency are limited in their ability to capture the moment-to-moment processing involved in visual word recognition. In the present study, the effect of word familiarity was initially investigated using an eye blink rate among adults and children. The probability of eye blinking was higher for familiar (real) words than for unfamiliar (pseudo)words. This counterintuitive pattern of results suggests that the processing of unfamiliar (pseudo)words is more demanding and perhaps less rewarding than the processing of familiar (real) words, as previously observed in both behavioral and pupillometry data. Our findings suggest that the measurement of eye blinks might shed new light on the cognitive processes involved in visual word recognition and other domains of human cognition.

## Introduction

In the field of reading research, there is broad agreement that efficient word recognition is crucial for reading comprehension (Hoover & Gough, 1990; Perfetti, 1985; Perfetti & Helder, 2022; Share, 2008; Stanovich, 2000). The developmental trajectory by which the young reader advances from slow, laborious word decoding to fast, efficient word recognition has been a perennial subject in the scientific study of reading. Central to the study of reading acquisition is the understanding that visual word recognition is experience-based and an item-centered process (Castles & Nation, 2008; Nation & Castles, 2017; Share, 1995, 2008). This is because every printed word is unfamiliar at some point in reading development (Share, 1995, 2008). According to the self-teaching hypothesis (Jorm & Share, 1983; Share, 1995, 2008), in the course of repeated exposures, the process of phonological recoding (decoding) permits an unfamiliar word to become increasingly familiar, hence recognized more accurately, more quickly and more effortlessly (Kuhn et al., 2010; Pikulski & Chard, 2005). It follows that investigating the word familiarity effect in terms of differences between reading familiar words and unfamiliar words is likely to illuminate critical aspects of information processing involved in visual word recognition (Sereno & Rayner, 2003).

Behavioral differences in speed and accuracy between familiar and unfamiliar words (such as pseudowords) are well-established and supported by a voluminous and converging body of empirical evidence across a variety of tasks, populations, and writing systems (Forster & Chambers, 1973). However, these conventional measures may not fully capture the neural underpinnings of printed word processing (Carreiras et al., 2014). Furthermore, these two ubiquitous measures may not provide direct insights concerning the crucial issue of effort and efficiency in word recognition. In order to redress this lacuna, we recently reported the dynamic changes in cognitive effort involved in the course of word recognition by tracking pupillary responses. Pupillometry – the measurement of task-dependent pupil dilation has been found to be a sensitive and reliable measure of cognitive effort and is now used in a variety of fields of investigation (Sirois & Brisson, 2014; van der Wel & van Steenbergen, 2018). Using pupillometry, we confirmed that the reading of unfamiliar words involves more cognitive effort than reading familiar words in both oral and silent reading modes among skilled adult readers and 4th–6th graders. These findings were evident in multiple measures, including greater overall pupil dilation, higher maximum (peak) dilation, and longer latencies to peak dilation (Shechter & Share, 2021).

Taking a wider perspective, Sereno, Rayner, & Posner (1998) and Sereno & Rayner (2003) sought to map the dynamics of the time course involved in visual word recognition. Using both eye movements and event-related potentials, they concluded that word processing activates higher cortical areas, with lexical processing beginning around 60 ms after the first fixation. Furthermore, based on the average fixation duration, they concluded that lexical processing occurs around 250 ms after stimulus onset, followed by a saccade and electrophysiological changes in both P300 and the N400 components assumed to reflect post-lexical processes.

In the present study, we aimed to continue the investigation of the temporal trajectory of the word familiarity effect (i.e., reading familiar versus unfamiliar (pseudo)words) with a temporal measure. As in other pupillometry studies, in our previous investigations (Shechter et al., 2022; Shechter & Share, 2021), we had to deal with the methodological challenge of eye blinks. That is a rapid closing and opening of the eyelid that causes missing time windows in the pupillometry data. It has already been suggested that the occurrence of eye blinks is not random but rather dependent on task characteristics (Stern et al., 1984), such as cognitive load (Fukuda, 2001). Interestingly, eye blink rate (EBR) is strongly associated with dopamine release (Jongkees & Colzato, 2016; Karson, 1983). That is, the more dopamine is released, the more eye blinks will be observed. Dopamine release is strongly associated with reward-driven behavior and cognitive flexibility, and therefore, an increment of EBR might be observed in tasks that will involve rewards (Di Chiara & Bassareo, 2007; Slagter et al., 2015) and cognitive flexibility (Dreisbach et al., 2005). Whereas reading unfamiliar (pseudo)words may require more cognitive effort (Shechter et al., 2022; Shechter & Share, 2021), reading familiar words might cause a positive reward. Examination of EBR as a result of the reading of (un)familiar words might answer this question.

In addition to the association between eye blinks and the reward system (Di Chiara & Bassareo, 2007; Slagter et al., 2015), it has already been suggested that eye blinks are strongly associated with task difficulty and mental effort (Maffei & Angrilli, 2018) in general. It means that the more difficult and demanding the task is, the fewer eye blinks will be observed. Therefore, in line with both behavioral and pupillometry findings, EBR might also provide us with evidence of the mental difficulty of reading (un)familiar words. That is, for familiar words, we expect less mental effort, and therefore, more eye blinks are predicted compared to unfamiliar (pseudo)words.

Another interesting association of eye blinks is with the release of resources used in stimulus-related cognition (Ohira et al., 1998). That is, once the stimulus is well-recognized, EBR will increase. Therefore, we expect that familiar words, which are easier to recognize, will cause more eye blinks than unfamiliar (pseudo)words that might be more difficult to recognize.

Employing the EBR method for investigating visual word recognition processes such as the word familiarity effect offers a unique source of potentially converging evidence –an essential aspect in every scientific endeavor. As opposed to the standard behavioral measurements of response accuracy and speed, EBR provides a temporal measure of processing. Furthermore, since participants are generally unaware of their blink rate, this measurement reflects an involuntary response. Hence, EBR appears to reflect a relatively “pure” measure of cognitive processing which is less likely to be susceptible to strategic processing as in the case of speed-accuracy tradeoffs involved in voluntary decision-making and response production. EBR may also provide insight into early visual information processing compared to pupillary responses which are characterized by slow response times. Finally, the measurement of EBR is likely to be easier to implement and analyze compared with other physiological measurements (such as ERP and pupillometry). For example, Soukupova and Cech (2016) have suggested that it is possible to detect EBR online using a webcam in experimental paradigms). Hence, we propose that this easy-to-implement approach may provide us with information about basic cognitive processes that have promise for research in many cognitive domains.

## The current study

In the present study, we aimed to examine the temporal trajectory of EBR when participants were asked to read aloud familiar and unfamiliar words. This is the first study to look at eye blinks as a tool for studying visual word recognition. In line with previous studies that examined the temporal trajectory of EBR (Siegle et al., 2008), we examined differences between the investigated conditions, as is frequently done in pupillometry studies (Hershman et al., 2022). Specifically, participants (adults and children) were asked to read aloud familiar and unfamiliar (pseudo)words. This kind of examination might provide us with new insights into the cognitive processes involved in visual word recognition.

## Materials and Methods

### Participants

The data in the current study was based on our previous work (Exp.1 and Exp. 3 in Shechter & Share (Shechter & Share, 2021)). Specifically, two age groups were examined. An adult sample consisting of 34 university students (27 females; mean age 27), and a sample of 34 fourth-to-sixth grade children (19 females; mean age 10) including 10 fourth graders, 11 fifth graders, and 13 sixth graders. The sample size for each age group was determined on the basis of a prior power analysis with power set at 0.80, an alpha of 0.01, and an intermediate effect size (f) of 0.25. Subjects were native Hebrew speakers, with no reported past or present reading disabilities and/or attentional difficulties and had normal or corrected-to-normal vision (for full details, see Shechter and Share (Shechter & Share, 2021)). The current experimental protocol was reviewed and approved by The Ethics Committee of the Faculty of Education of the University of Haifa (Approval no.18/427), based on the relevant ethical guidelines and regulations. A voluntary informed consent form was signed by each participant and each participating child’s legal guardian before the actual participantion.

### Stimuli

Participants were presented with 80 Hebrew unfamiliar letter-strings (pseudowords) and 80 familiar (real) words.^1^ The target stimuli were presented in four blocks. Each block contained 40 stimuli divided into two examined conditions: 20 pseudowords and 20 real words. These conditions were matched phonologically, morpho-phonologically, for length, and for luminance. In order to provide an ecological range of word frequencies and minimize possible strategic artifacts during task performance, each block also contained filler words representing a variety of parts of speech and word lengths (20 items for the adults, and 10 items for the children). The viewing distance was 57 cm, such that the target stimuli subtended a visual angle of 1.11° to 1.61° for height and 2.21° to 4.82° for width. All stimuli were centered, displayed in white (RGB = 255, 255, 255) on a gray background (RGB = 128, 128, 128). For further information regarding the way we created the stimuli list see Shechter and Share (2021).

#### Procedure

Subjects were individually tested in a dimly lit sound-reduced room at the Edmond J. Safra Brain Research Center for the Study of Learning Disabilities at the University of Haifa. They were required to read out loud the presented word which disappeared automatically. The trial sequence was the same for the two samples, except for one change: stimulus presentation. Stimuli were displayed for a longer duration for children (4,700 ms) compared to adults (3,300 ms). Reading pronunciation errors were manually documented by a tester.

### Apparatus

An Eyelink 1000 Plus (SR Research, Ontario, Canada), an infrared eye tracker with a sampling rate of 1000 Hz, was used to record the pupillometry data. To maintain intact measurement of pupil size, calibration and validation preceded each block. Furthermore, the participants were asked to keep their eyes fixed on the center of the screen, and not to shift their gaze position during the entire session.

## Results

### Pre-processing of pupillometry data

Pupil data were processed using the CHAP software (Hershman et al., 2019). First, pupil data were extracted from the EyeLink (pupil size in arbitrary units). Then, we removed outlier cases with Z-scores larger than 2.5. Z-scores were calculated based on the mean and standard deviation for pupil dilation for each trial. Next, we calculated the percent of outlier values for each participant in each trial and excluded from analysis trials with more than 20% missing values. We also excluded trials with incorrect or missing responses. We defined a minimum number of 20 valid trials for each condition, so no participant was excluded from the analysis. This pre-processing of pupil data eliminated 14.3% of trials on average (see full information about the exclusion rate in each condition in each group in the Supplementary material reported in Shechter et al. (Shechter et al., 2022)).

Next, we detected eye blinks by using Hershman, et al.’s (2018) noise-based algorithm. In general, eye-tracking devices often produce noise and the noised-based approach uses this noise to more accurately detect the start and end of eye blinks. When the eye is closed, the noise signal disappears and when the eye is open, the noise signal reappears. By identifying these moments in time, the algorithm accurately defines the onset and offset of each blink (Hershman et al., 2018).

Then, for each condition, for each time point, the probability of eye blinking was calculated (i.e., the number of eye blinks on the time point divided by the number of valid trials).^2^

### Eye blink rate

In order to examine the temporal differences among the conditions for each age group separately, we used the approach of Hershman and Henik (Hershman & Henik, 2019) for the analysis of pupillometry data. For each age group, we ran time-series analyses in terms of Bayesian paired sample t-tests between the conditions of interest. Specifically, we compared the eye blink rate of real-word trials to those of pseudoword trials. We used a default Cauchy prior width of *r* = .707 for effect size on the alternative hypothesis (Rouder et al., 2012). The Bayes Factor (BF) threshold was 3, a value that represents “moderate” evidence (Jeffreys, 1961).

**Figure 1:**
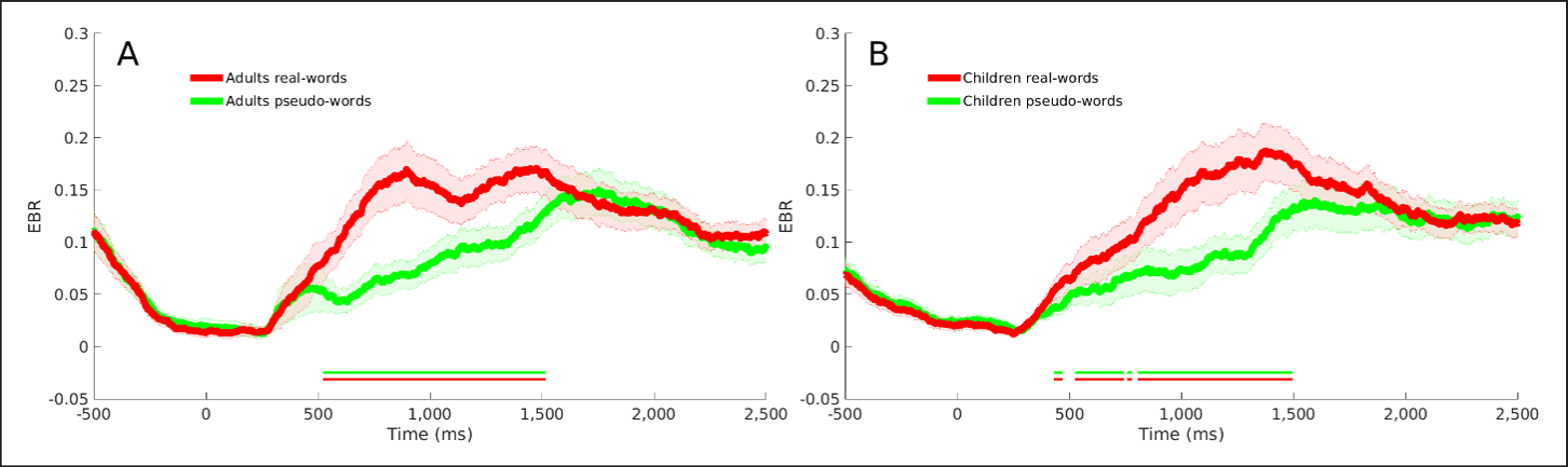
Eye Blink Rate (EBR) for real words (red) and pseudowords (green). **(A)** adults. **(B)** children. *Note*: The shaded areas represent one standard error from the mean. The double horizontal lines indicate meaningful differences (i.e., *BF*_10_ ≥ 3) between conditions.

As reflected in both adults (Figure 1A) and children (Figure 1B), the EBR before the stimulus onset and immediately after the stimulus onset was minimal (the probability of eye blinking was less than 5%). After about 270 ms following the stimulus onset (for adults: 275 for real-words and 260 ms for pseudowords; for children: 250 ms for real-words and 286 for pseudowords) when the EBR reached the minimal value (less than 1%), boosts of eye blinks started. The boosts reached a maximal value (adults: real-words: 29% & pseudowords: 22%; children: real-words: 28.4% & pseudowords: 22.1%) after about 1,585 ms following the stimulus onset (adults: real-words: 1,303 ms & pseudowords: 1689 ms; children: real-words: 1,559 ms & pseudowords: 1,788 ms). Analysis of the observed maximal EBR values suggested that real words led to higher EBR values than pseudowords adults: *t*(33) = 4.324, *p-value* < .001, *BF*_10_ = 195.3; children: *t*(33) = 2.316, *p-value* = .027, *BF*_10_ = 1.88). Another analysis of the observed maximal EBR values suggested that real words tended to reach the maximal values earlier than pseudo words (adults: *t*(33) = 5.32, *p-value* < .001, *BF*_10_ = 2,847; children: *t*(33) = 6.242, *p-value* < .001, *BF*_10_ > 10^4^).

Temporal analysis of the adults’ data (Figure 1A), as well as analysis of the children’s data (Figure 1B), showed meaningful differences (*BF*_10_ ≥ 3) between real words and pseudowords. Specifically, the increment of EBR for real words was faster and more frequent than that for pseudowords. As reflected in Figure 1A, for the adult samples, meaningful differences were observed from about 530 ms after stimulus onset until about 1,500 ms post-onset. A similar pattern was also observed for children (Figure 1B). Specifically, meaningful differences were observed from about 440 ms post-onset until about 1,490 ms post-onset.

## Discussion

In the present study, participants (adults and children) were presented with familiar and unfamiliar (pseudo)words and were asked to read them aloud. In previous behavioral studies using the same design used here, differences have been found in speed and accuracy between these two conditions. These findings are well-established and supported by a voluminous and converging body of empirical evidence across a variety of tasks, and populations (Forster, 1970). Recently, we used changes in pupil size to examine the differences between these conditions in terms of the cognitive effort invested in word recognition (Shechter et al., 2022; Shechter & Share, 2021). These pupillometry studies confirmed a greater degree of cognitive effort for reading unfamiliar (pseudo)words compared to familiar words, among adults and children alike, in both oral and silent reading modes (Shechter & Share, 2021). Furthermore, we found that children invested more effort in reading than adults, as indicated by larger and sustained pupillary responses. However, in each age group, the faster readers demonstrated accelerated pupillary responses compared to slower readers, although both groups invested a similar overall degree of cognitive effort (Shechter et al., 2022). In the present study, we aimed to examine the effect of word familiarity by using changes in the temporal pattern of EBR.

First, our results suggested that the EBR reached a minimal value (less than 1 %) close to the stimulus onset. After about 270 ms following the stimulus onset, a boost of eye blinks was observed (for both adult and child participants). This time point (i.e., about 270 ms following the stimulus onset) has already been found in ERP studies using P300 and the N400 components that are assumed to reflect post-lexical processes (Sereno et al., 1998; Sereno & Rayner, 2003). In other words, our findings are in line with previous ERP studies (in terms of timing) suggesting that the initial processes of word recognition have been completed around 270 ms after the stimulus onset.

In addition, our results suggested that when the stimulus presented was a word, the EBR increased significantly more compared to the EBR when the stimulus was a pseudoword. This increment of the EBR is reflected in the maximal EBR value, in the latency to the maximal EBR, and in general, in the time window between 500–1,500 ms following the stimulus onset. Interestingly, in our experiment, this pattern was observed in both adult and child participants, suggesting that the observed pattern is robust and reliable.

In line with to the frequently reported behavioral (i.e., both RT and accuracy) data (as well as the pupillometry data) that frequently show more effort for unfamiliar words compared to familiar words, the EBR pattern suggested the same conclusion. Specifically, our results suggest more EBR for familiar stimuli than for unfamiliar stimuli. It has already been suggested that eye blinks do not occur randomly (Stern et al., 1984), but are often associated with cognitive load (Fukuda, 2001, 1994; Ohira, 1996) and information processing (Ichikawa & Ohira, 2004; Siegle et al., 2008). In contrast to Fukuda’s findings and in line with Siegle et al. findings (Siegle et al., 2008), we found evidence for more eye blinks for the less demanding task (as reflected in all our EBR parameters). One possible explanation for the observed pattern is that eye blinks are associated with the release of resources used in stimulus-related cognition (Ohira et al., 1998). Here, when participants easily recognize a highly familiar word, attentional resources are released. In contrast, when the stimuli were unknown (i.e., pseudowords), the stimulus required more time to process and recognize (at least, in orthographic/phonological terms).

Another possible explanation for the observed pattern is that eye blinks are associated with task difficulty and mental effort (Maffei & Angrilli, 2018). That is, the eye blink rate is expected to decrease during a difficult task compared to an easy or moderately easy task. In the present task, both behavioral and pupillometry data indicated that pseudowords are more difficult to process than real words (Shechter et al., 2022; Shechter & Share, 2021). Hence, fewer eye blinks are expected during the processing of pseudowords than real words.

Another interesting possible explanation for the observed pattern is that eye blinks are associated with dopamine release. It has already been suggested that the more dopamine released, the more eye blinks are observed (Jongkees & Colzato, 2016; Karson, 1983). Dopamine release is strongly associated with the reward system (Di Chiara & Bassareo, 2007). Therefore, during exposure to real words (compared to pseudowords), productive processing of the stimuli leads to successful recognition of the stimuli. This meaningful recognition might activate the reward system that is reflected in increased dopamine release, which is observed in higher EBR.

In general, children are characterized by slower reading rates than adults (Shechter & Share, 2021). Hence, a different temporal pattern might be expected. However, the observed pattern suggests that EBR is probably associated with a basic cognitive stage of word familiarity that is similar for both skilled and developing readers. Similar to P300 and the N400 components (Sereno et al., 1998; Sereno & Rayner, 2003), our results suggest that EBR might be used as an indicator for “pure” non-strategic word recognition. Further studies that will examine the temporal pattern of EBR for special populations, such as disabled readers, might expand our knowledge about the cognitive processes involved in visual word recognition.

We compared conditions using series of Bayesian t-tests. This approach is useful for the detection of differences between two conditions of interest. But it might not sufficient when one is interested in interaction effects. This difficulty may be solved by series of temporal (Bayesian) ANOVA tests or by other advanced statistical approaches. A commonly used approach is the linear mixed-effects (LME) analysis (Mathot et al., 2014). This approach might be useful in relatively complex experimental designs (e.g., for complex interactions). Another approach is a non-parametric cluster-based paired sample t-tests (Knapen et al., 2016) that is similar to analyses of blood-oxygen-level-dependent (BOLD) response in functional magnetic resonance imaging (fMRI) studies. As far as we know, the current research is the first to apply a temporal perspective in the field of EBR. We hope that this paper will encourage researchers to improve the definitions, norms, and procedures of temporal analysis.

## Summary

In the present study, we aimed to examine the use of EBR as a tool for shedding light on the cognitive processes involved in visual word recognition. Participants (adults and children) were presented with real familiar words and unfamiliar (pseudo)words. Whereas analysis of behavioral data as well as pupillometry data showed slower responses as well as more pupil dilation for pseudowords compared to real words, the temporal pattern of EBR revealed the opposite pattern. Specifically, close to the stimulus onset, EBR was minimal. About 270 ms after the stimulus onset (the time-point that is associated with word recognition among skilled readers) (Sereno et al., 1998; Sereno & Rayner, 2003), a boost in eye blink rate was observed. More EBR was observed for real words than for pseudowords, consistent with previous interpretations of both behavioral and pupillometry outcomes. This pattern is reflected in the maximal EBR value, in the latency to the maximal EBR, and in general, in the time window between 500–1,500 ms following the stimulus onset. This pattern might suggest that the processing of pseudowords is more demanding and perhaps less rewarding.

## Data Accessibility Statement

The code that was used to analyze the pupillometry data is available at: http://in.bgu.ac.il/en/Labs/CNL/chap. All experimental data can be found on the OSF (Open Science Framework): https://osf.io/hk4yq/.

This study was not preregistered.

## Footnotes

Although the terms familiar and unfamiliar in the word recognition literature are typically used when comparing high frequency and low frequency words, we retain these terms but use them in a broader, developmental sense (see Share, 2008), by noting that every word (even high-frequency words like ⟨do⟩⟨the⟩ and ⟨to⟩ begin life (in the novice reader’s head) as an unfamiliar string of symbols. With reading experience and repeated exposure, they eventually become fully-specified orthographic representations. Along this developmental continuum (unique to each reader, because no two readers read the identical materials), we see no functional difference between an unfamiliar letter string that is a true pseudoword and a string that has never before been encountered (e.g., ⟨bice⟩ and ⟨tringle⟩) are real English words found in good dictionaries but few English readers (even highly literate ones) would be able to say if these are pseudowords or real words. Therefore, pseudowords and unfamiliar real words are functionally identical – the only difference is that with pseudowords the experimenter can be certain that the string is indeed unfamiliar.

The probability of eye blinks was calculated using a designated feature in CHAP software (Hershman et al., 2019).
